# Social representations of malaria in the Guna indigenous population of Comarca Guna de Madungandi, Panama

**DOI:** 10.1186/s12936-017-1899-4

**Published:** 2017-06-15

**Authors:** Lorenzo Cáceres, José E. Calzada, Amanda Gabster, Josue Young, Ricardo Márquez, Rolando Torres, Margarita Griffith

**Affiliations:** 1 Department of Medical Entomology, Gorgas Memorial Institute for Health Research, 0816-02593 Panama City, Republic of Panama; 2 Department of Parasitology, Gorgas Memorial Institute for Health Research, 0816-02593 Panama City, Republic of Panama; 3 Department Proteomic and Genomic, Gorgas Memorial Institute for Health Research, 0816-02593 Panama City, Republic of Panama; 4 Universidad Latina de Panamá, Panama City, Republic of Panama

**Keywords:** Indigenous, Guna, Madungandi, Comarca, Social representation, Malaria, Perception, Practice, Tradition, Panama

## Abstract

**Background:**

The purpose of the study was to recognize the social representation of the Guna indigenous population by identifying cultural elements related to malaria, in order to create an intercultural approach to any health intervention to control and prevent the disease.

**Methods:**

This qualitative study has an anthropological focus that used participant observation, informal conversations, focus groups, interviews, in-depth interviews with key actors. Analyses included review, classification and categorization of interviews.

**Results:**

Malaria within the Guna culture is in harmony with several cultural factors, such as magic, religion, beliefs, myths, and nature. The health system must include these factors in its intercultural approach to ensure the sustainability of anti-malarial intervention measures. Until this is not properly addressed, the Guna population of Madungandi will remain a permanent source of risk for malaria transmission in this region and for the rest of the country.

**Conclusion:**

The findings of this study contribute new variables that can facilitate an intercultural approach to improve the perception of malaria in the indigenous population of Comarca Guna de Madungandi, Panama.

**Electronic supplementary material:**

The online version of this article (doi:10.1186/s12936-017-1899-4) contains supplementary material, which is available to authorized users.

## Background

Health concepts have cultural and historic variations [1]. In indigenous populations, health is one of several concepts of an indigenous society, expressing a dynamic relationship with an equilibrium between inseparable components, of what it is individual (physical, mental, emotional, spiritual), and what it is collective (ecological, political, economic, cultural, social, spiritual). This integral health concept includes the biological, psychological, social, and spiritual wellbeing of a person and of the community in equal conditions [2].

Health can be defined in specific forms of order and action (individual or collective), and intervention in order to promote, maintain and restore the health of a population [3, 4]. In general, the definition of health and disease will condition an attitude to treatment and cure priorities. Research seeks to discover the knowledge, attitudes and practices that identify the feelings of populations: what they do when they become ill and what is their attitude to certain practices that are intended to prevent and/or cure a disease. Important studies have been carried out which help in understanding the relationship between health and disease. Major contributions are required from the social sciences to understand the complexity of the benefits that health brings to communities [5].

Social and cultural aspects that are related to malaria and its prevention and treatment, for example, housing, poverty, education, knowledge, attitudes, and practices of a population, help explain and thereby improve implementation of designed interventions [6]. Although several analyses point out the cause of a health situation of indigenous populations, the solution places emphasis in health services to concentrate exclusively on cultural characteristics of such populations. It is fundamental to promote actions that produce intercultural relations, which allows dialogue and respect in any approach to intervention [7].

The main problems that indigenous populations face, and which are a vehicle for malaria transmission and other metoxenous diseases, include poverty, analphabetism, unemployment, migration, limited access to public services, environmental degradation, changes in the pattern of settlement and life dynamics, pressure on frontiers of colonization and development, and social exclusion from the development proposals of countries [8, 9]. Malaria is present in specific social territories with unique historic and social dynamics, particularly in poorer communities that have minimal sanitation [10–12]. A lack of permanent health services for indigenous populations leads to malaria morbidity and mortality.

Indigenous populations are the most affected segments of society in Panamá, with abysmal differences in health indicators compared to the national average, such as maternal mortality indexes, malnutrition, and high rates of morbidity due to preventable causes. The limiting factors of access to health services due to geographical, economic and cultural barriers are problems that affect the majority of indigenous populations. These factors present a true challenge to find health solutions that accord to cultural characteristics, such as language, traditions, beliefs, and cosmovision. The indigenous peoples, who make up the more critical extreme of social exclusion in Panamá, account for the highest levels of respiratory diseases, such as tuberculosis, and vector-borne diseases, such as malaria [13]. Such poverty is described as “abysmal” by the *Banco Mundial*, and “massive and profound” by the National Government [14].

This present study aims to unravel the complexity of malaria within the cosmovision of the Guna indigenous population, and focuses on the need to develop investigations and processes of qualitative nature, to look not only at the epidemiological aspects of malaria, but also at the perceptions of the indigenous populations to this disease. The objective was to determine how cultural representations and practices can improve the monitoring, prevention and control of malaria transmission in the Guna indigenous population of Comarca (‘Comarca’: special administrative region that serves as reservations for Panamanian citizens of native American descent) Guna de Madungandi, Panamá. The results of this study may contribute to an understanding of inter-relations existing between malaria, culture, environment and social development in the indigenous Guna population. The study also contributes to the National Malaria Control Programme of Malaria (NMCP) of the Ministry of Health (MOH), in defining a methodological approach with an intercultural focus to intermingle the language and cosmovision of the Guna population of Madungandi, respecting them, and making possible an ethnic development for an improved quality of life.

## Methods

### Site of the survey

**Fig. 1:**
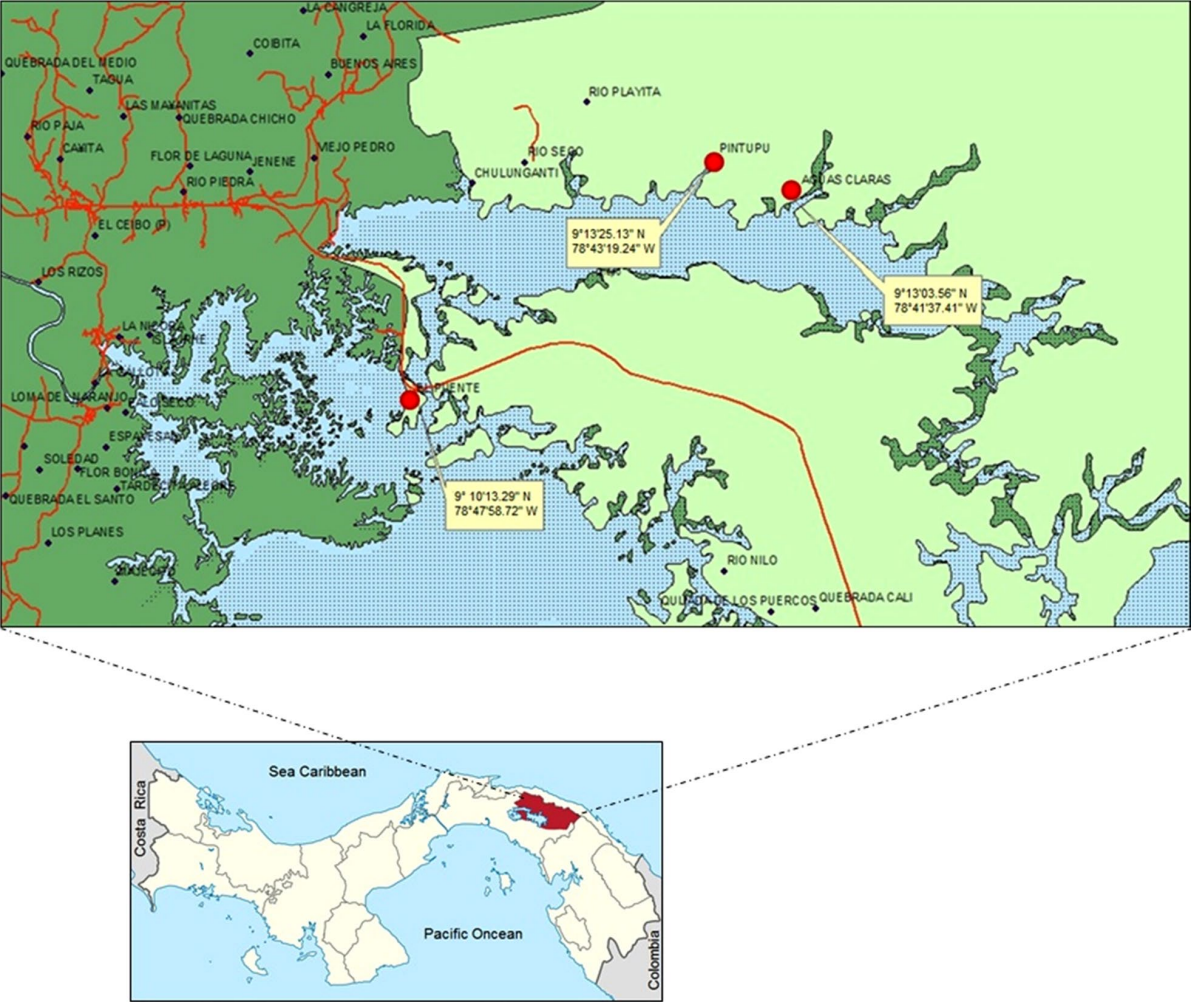
Geographical location of communities Icanti, Akua Yala and Pintupo in the Comarca Guna de Madungandi, Panama

The investigation took place in the Comarca Guna de Madungandi, located in eastern Panamá. Its surface is 2318.8 sq km, and it is situated in proximity of Bayano Lake. The population is 4271 persons [15]. The communities selected for this survey were: Icanti or Aguas Claras [9°13′03.56″N; 78°41′37.41″W and 72 m above sea level (masl)] with a total of 78 dwellings and 820 inhabitants; Pintupo or Nuevo Espavé (9°13′25.13″N; 78°43′19.24″W and 67 masl) with a total of 28 dwellings and 225 inhabitants; and, Akua Yala or Puente Bayano (9°10′13.29″N; 78°47′58.72″W and 70 masl) with a total of 48 dwellings and 274 inhabitants, all situated in the margins of the Bayano Lake (Fig. 1).

### Study design

A qualitative, descriptive and exploratory design was applied. The investigation was approached with methodological strategies with an anthropological focus, which combined participant observation, informal conversations, focus groups, and in-depth interviews directed to key actors.

### Population study

The tools of participant observation and informal conversations produced a sample of 1319 persons, chosen by a random procedure from the communities of Icanti, Pintupo and Akua Yala [16]. In-depth interviews and focus groups were directed to key actors and informants previously identified in the three selected communities.

### Ethical considerations

The protocol of the project was evaluated and approved by the National Commission of Bioethics May 2012. A presentation of the survey and study objectives were made to the traditional authorities of the General Congress of the Comarca Madungandi (GCCM), who approved its performance. The survey was also presented to MOH at national, regional and local level, to identify the support group. For the development of the interviews, each participant was informed verbally and in writing, about the nature of the survey. Informed consent was signed by those interviewed to record and take notes before a witness, of which the investigation team and the participant kept a copy. Before performing interviews the objectives of the survey were explained to each participant, giving them assurance of confidentiality of the recorded information.

### Survey approach and social connotations

In the development of this survey, several phases were followed to achieve the investigation goals. The first phase consisted of a request to the GCCM for a permit to develop the survey; once the project was delivered, it went for a revision to the Technical Commission of the GCCM, which took 6 months to grant the permit to initiate the survey. The second phase was to inform the *sahilas* and local congresses of the selected communities, to hand in the visit timetable, and obtain the permit to initiate the survey in such communities. A third phase was to select key informants in each community; some were persons recommended by the officers of the NMCP, due to their path of cooperation with MOH. These key informants provided great support in organizing the focus groups and the selection of persons to be interviewed. Finally, the fifth phase was the formation and selection of the focus groups, in-depth interviews and key informants.

### Participant observation

Through day-to-day experience living with the subjects being surveyed, it was learned the significance of many symbols and cultural practices in the communities, creating a direct connection with them, which allowed the openness that needed to obtain data. Participant observation was performed during a total of four tours to each community, of approximately 5 days each tour, during which all participants involved were interviewed. Informal conversations were of non-structured dialogues with the survey subjects, carried out in a sporadic fashion without previous planning, and were a complement to participant observation.

### Focus groups

Focus groups were made up of 17–19 participants, a monitor who directed the conversation, and a facilitator to obtain data, and were of approximately 45 min duration. There were nine focus groups, each one comprised youngsters, women, men, teachers, and members of organized groups. All the focus groups were organized with the support of a Guna translator, following the guidelines of questions developed by the investigation team. These group discussions were recorded to obtain accuracy at the stage of analysis of the speeches at semantic and syntactic level, gathering data in categories.

### In-depth interviews

Key members were identified within the communities, which were traditional doctors, *sahilas*, community leaders, health workers, members of the Guna Organization of Madungandi, (GOM), and persons who at some time had been malaria patients. A total of ten in-depth interviews were performed in the three communities selected for this survey. The in-depth interviews allowed to determine the more common practices for the treatment of malaria and question in detail some specific aspects, for example, the meaning of the rituals to treat malaria (Table 1).

**Table 1:** Number of focus groups and interviews conducted on cultural aspects of malaria in the Guna population of Madungandi, Panama

Focus groups by location and number of participants	Key informant interviews	Interviews with health officials
19 women Pintupu 9 women of Akua Yala 10 women of Icanti 9 men of Pintupu 6 men of Akua Yala 11 teachers Icanti 7 youth Icanti 9 young Akua Yala Group 16 young sports Akua Yala	First Sahila, Icanti community Secretary of the Congress Icanti Second Sahila Pintupu community First Sahila community of Akua Yala President of the GOM Administrator GOM	National Coordinator of the NMP NMP Coordinator of Health Region East Panama NMP officials Manager of the Health of Akua Yala

### Data analysis

For the analysis of the information, a dynamic and integrative process of revision, organization, classification, categorization and interpretation was performed. Once the information was classified, discursive analysis of the language and analytical inferences was performed to arrive at an understanding of the studied phenomenon. An analytical matrix of contents was used in which speeches and activities of the observed subjects were classified [7–19]. The analysis unit was composed of testimonies provided by members of the communities of the selected sites. Descriptive codes were used for actual expressions by the persons studied to preserve data veracity and illustrate the interpretations and conclusions of the investigation. The methodology utilized for the presentation of this survey used the categorical system [20, 21].

## Results

### Background of the malaria programme in regions with indigenous populations

**Fig. 2:**
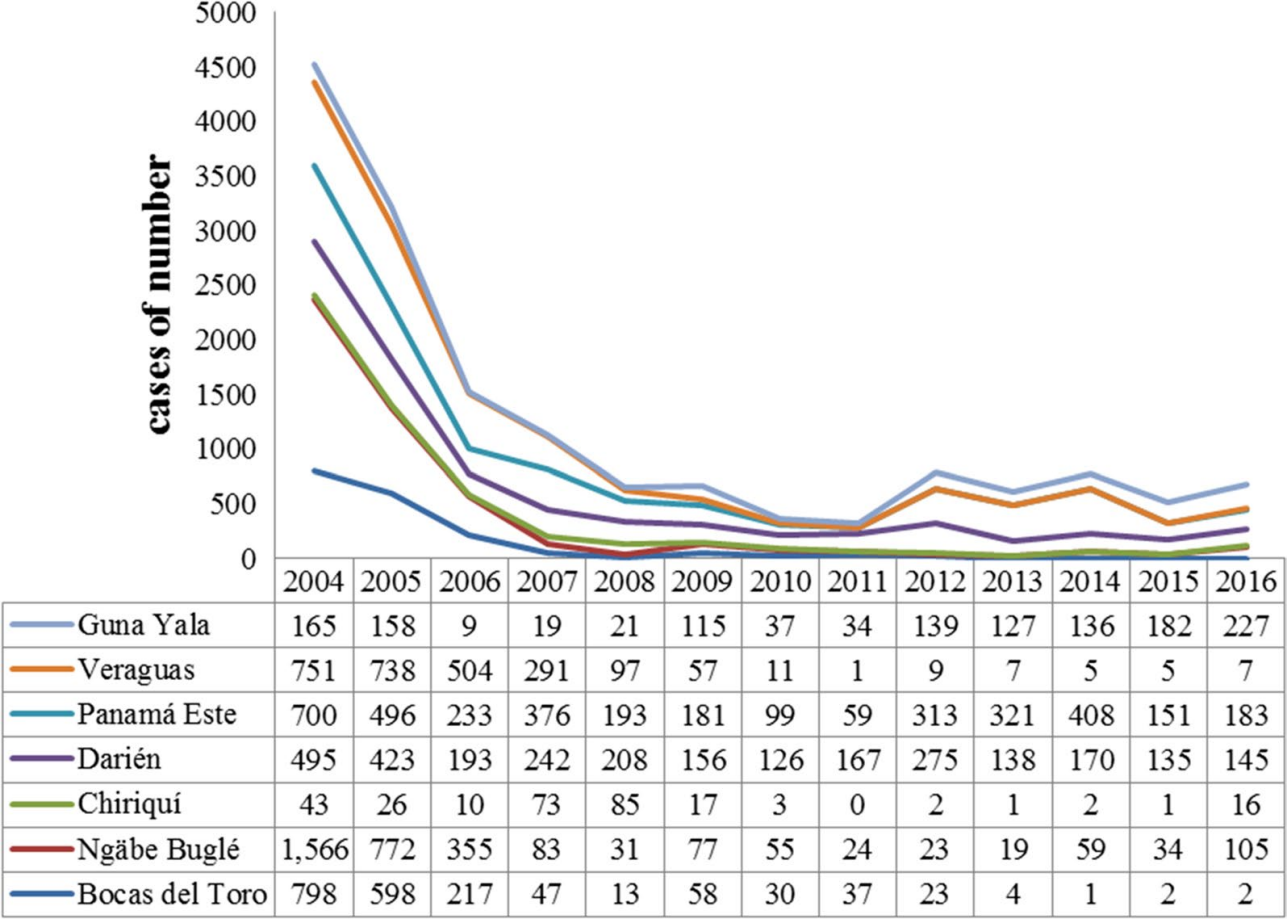
Epidemiological behaviour of malaria in regions with indigenous populations in Panama, 2004–2015

The analysis of malaria in the last 30 years in Panamá established the geographical distribution and endemic/epidemic transmission patterns of the disease. The pattern of endemic transmission reaches areas with indigenous populations in the provinces of Bocas del Toro, Chiriquí, Veraguas, Darién, the indigenous comarcas: Emberá-Wounaan, Ngäbe-Buglé, Guna Yala, Wargandí, and Madungandi. During the period 2004–2016, a total of 17,812 cases of malaria were registered, with an average of 1370 cases diagnosed annually in the poor, rural and mainly indigenous areas. During this period, a parasitic incidence was registered with an annual average of 0.4/1000 inhabitants, an average morbidity rate of 40.6/100,000 inhabitants, registration of eight deaths and 90.0% of malaria cases were by *Plasmodium vivax.* In the regions with an indigenous population, a total of 15,729 cases were registered, corresponding to 88.3% of the total malaria cases diagnosed at national level during this period (Fig. 2).

### Survey results and social connotations

The presentation of the results of this survey are organized in the following six sections, which provide important evidence and cultural information about the associated social connotations of malaria in the Guna indigenous population of Comarca Guna de Madungandi.

### Health, disease and death within Guna culture

According to ancient tales of the Guna, *Pap Dummand,* the supreme God, created in the beginning the whole universe: plants, animals, *Olobilipiler,* (man), and *Oloporsubi,* (woman). But the men and women created by *Pap Dummand* did not take care of nature and carried out incorrect activities, and the Earth was punished with maladies, one of these being the Father of the Diseases, named *Kachuka.* He scourged the Earth with terrible diseases and epidemics which killed a lot of people.

**Fig. 3:**
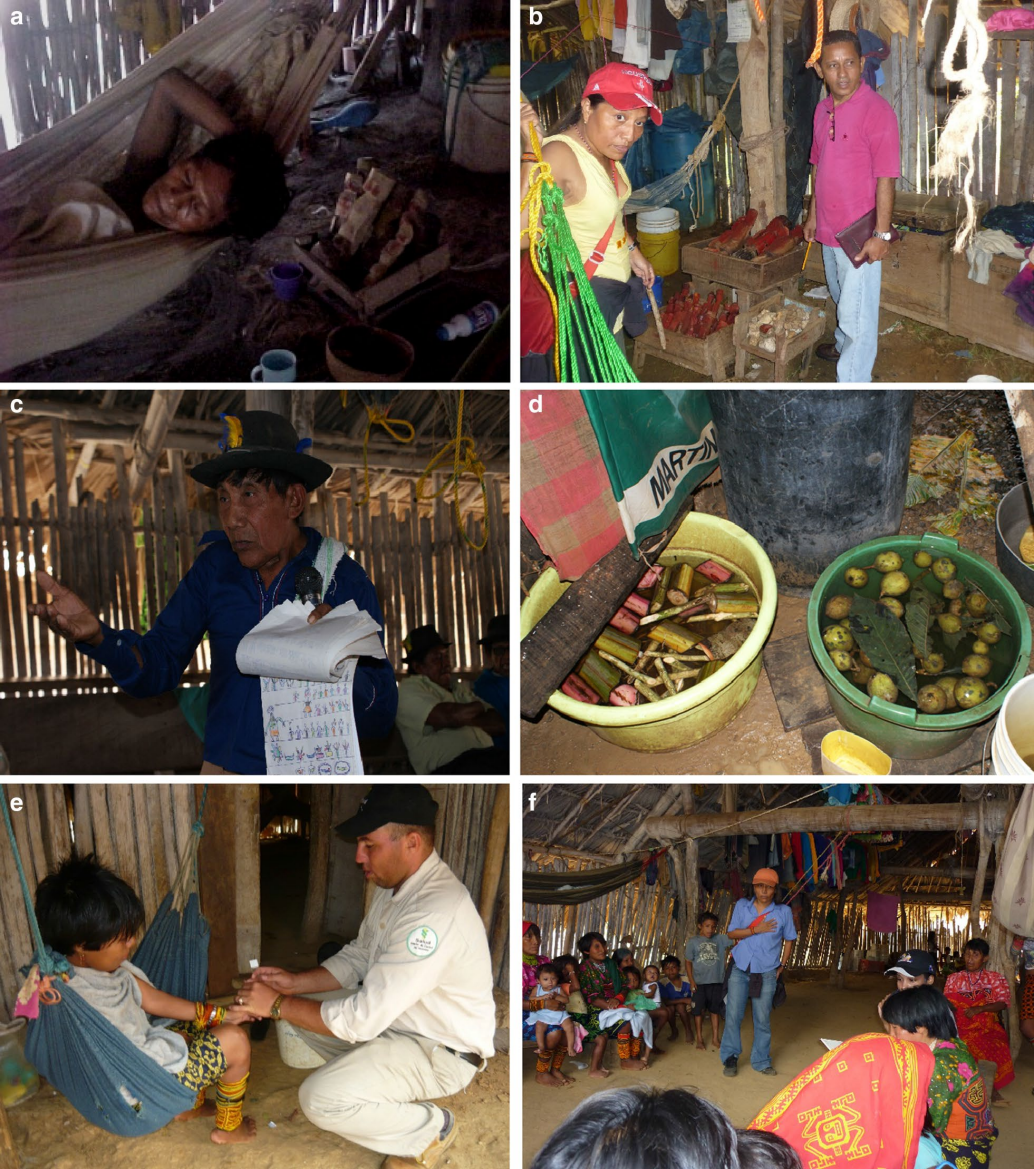
Malaria has a very close relationship with Gunas customs, traditions and cultural practices. **a** Malaria being treated with traditional medicine and the use of “*nuchos”*. **b** “*Nuchos”* and “Stones” used by Guna to prevent disease. **c** Traditional medical community of Akua Yala, Madungandi. **d** Medicinal plants used by Guna in healing baths. **e** Woman of Icanti, Madungandi. **f** Focus group of women from Icanti, Madungandi

For the Guna people of Madungandi, health is the result of integration of social-magic and biological factors. A lack of balance in nature caused by disobedience of the social rule or negligence in hygiene measures causes disease. Those who bring the disease are spirits and the only way to restore order is to be at peace with them. This restoration is obtained through the traditional medicine (Fig. 3).

*“We have sacred places called Kalu. The spirits live there. When they get mad, then the epidemics appear. People start to die. The* sahila *calls the* Absogedis *and he tells the community if we have to smoke the pipe”.* GOM Administrator

The binomial of health/disease within the Guna indigenous cosmovision of Madungandi is integrated by a series of ideas, concepts, beliefs, myths, and procedures, which in the majority of cases is not explained in a scientific way but by spiritual forces; there are protective spirits as well as malign ones that cause disease. In order to cure the diseases, Gunas, who from infancy have been endowed with special gifts to cure, are named *Neles or Innatuledis.* Above all nature spirits, there is the supreme being, *Pap Dummand*.

*“For us the Gunas, everything in nature has a spirit, the trees, the plants. The Earth too. She is our mother*-*Earth and she must be protected. When we soil it, or mistreat her, then the spirits send us diseases as a punishment. Only the see*-*ers have the faculty of contacting those spirits to know what to do*”. Informal conversation, woman from Icanti.

The traditional, non-scientific knowledge which explains the origin of the human being, as well as his relation with mother-Earth and the spirits that manage it, is named the indigenous cosmovision.

### Perceptions referring to malaria

In the focus groups, malaria was approached by two initial questions that were proposed to the participants:

*What are some of the main problems that face this community?*

*What are some of the main diseases that afflict this population?*

Health problems more frequently identified were diarrhea, vomiting and colds. Among the main problems identified were lack of water and medicines. Malaria was not identified as a health problem, but once asked them directly if malaria was a health problem, participants answered positively.

*Sahila* of Icanti, indicated:

*“About malaria, I can tell you the following: malaria and cholera appeared strongly when the Bayano area was inundated and the lake was born. With this happened many diseases showed up, but the ones feared the most were malaria and cholera. These diseases come and go.”* Interview, Icanti *sahila*.

In general, women were mainly interested in malaria since they remain at home taking care of the children. As one related:

*“Men are never at home, they are always travelling or in the woods. We are the ones who must take care of the home and take care of the children if they become sick. We do want to know more about how to treat malaria. I feel that this meeting is going to be of interest for us, because we are mothers and women carrying the weight of the home*”. Focus group, women from Pintupo.

The men of Pintupo knew where the disease comes from, but they did not know adequate ways to prevent it, as was indicated by one of them:

*“As I understand, malaria is produced by a mosquito, but mosquitoes have always been here. Ever since our grandfathers, there have been mosquitoes. They will always be with us. It is impossible to do something about it*”. Focal group of men from Pintupo.

### Use of traditional medicine

For the Guna population of Madungandi, malaria is caused by external spirits and/or the lack of equilibrium that could exist in a determined moment between nature and man. Here the importance of traditional medicine is seen, since through it the population manage to restore equilibrium and obtain health. Traditional medicine, according to the Guna, is a holistic science that embodies physical elements: social, environmental, spiritual, and territorial. Curative therapy includes knowledge of medicinal plants, sacred sites, communication with spirits, and other biological resources. It was expressed by one of the *sahilas* interviewed:

*“When our Father Baba left us, he gave us something that is ours, the nature, the plants, that have leaves, branches, roots for us to use as our medicine. It is what our Gods gave us. Thousands of years ago he left us all that he built for us”.* Interviews, second *sahila* of Pintupo.

The ethnomedical system of the Guna from Madungandi, as well as the other ethnic groups of Panama, have specialists divided in several disciplines to treat diseases through a direct relationship with the spirits:

*Nele* is leader of the ethnomedical system and is the name of the Guna who has the power to contact protective spirits and cure diseases. Differing from the Neles, the *Innatuledi* does not communicate with the spirits, and only knows about the curative powers of plants and bones from animals. *Absogedis* Gunas are specifically dedicated to prevent and fight disease. In order to perform rituals, they create *nuchos* (wood figures) to whom they sing so that the good spirits in the *nucho* come out and go after demons who cause diseases.

One of the traditional doctors interviewed explained the above as follows:

*“We have our own doctors. The most important one is the* Absogedis*. He is like a specialist. He is called when diseases are present. The* Absogedis *come into contact with the spirits and tell where the malady is. Another important doctor is the* Nele*. Thereare almost no* Neles *nor* Absogedis *in these communities nowadays. Not everybody can be a* Nele *or* Absogedis*. The* Innatuledi *are the botanics. There are a lot of them. In this community there are about seven. We do not work with the spirits. We work with the plants.”* Interview, traditional doctor from Pintupo.

Another traditional doctor, in his interview, informed about the existence of the Kandur:

*“There is a variety of specialties, like in the case when there are diseases, we can count on many* Igargan *(persons specialized in the chant of recovery of the spirit of the Earth). Equally, we have the* Nele *(a person who diagnoses diseases through sleep and use of aromatic smoke, having as the main ingredient, the cocoa seeds. The* Kandur *is the specialized priest in the ritual of the* chicha brava *and other knowledge that is still practised in our Comarca.”* Interview, traditional doctor from Icanti.

A fundamental part of traditional medicine are the medicinal oral Guna chants of *Igargan*, that are the curative and therapeutic chants raised during treatment and which have the strength to cure diseases that are generally produced by the spirits. The chants are executed by the *sumaquet,* the specialist of the chant (Table 2). The curative processes utilizes figures carved in wood, called *Nuchus*. The *Nuchus* have inside them the spirits of great *Neles* that protect people from diseases.

**Table 2:** Some therapeutic songs and their use in healing therapy by Guna population of Madungandi, Panama

Song	Traditional uses
Nia Igar	This song is used against madness and in patients with schizophrenia
Sia Igar	This song is performed using cocoa and serves to rescue the soul
Gabur Igar	Its singing with chili pepper, used to rescue the soul and to smoked the home from the spirits
Muu Igar	Singing for treating women for difficult births

### Traditional practices to cure malaria

Vector control faces implementation difficulties within communities when the ritual of *Waa Ued,* or ‘Smoking of the Pipe’, is celebrated. According to data obtained from this ritual, the Guna have sacred sites called *Kalu* (outer spaces where forces of nature live alongside spirits and gods), located in the mountains, rivers, and in entire nature. When one of these sites is desecrated, the wrath of the spirits is awakened, and epidemics are sent to cause death to several members of the community. In order to calm this wrath, the men of the community must get together to smoke the ‘Pipe’, which is a type of tar, in order for the community to be healthy again.

The ritual of Smoking of the Pipe can be realized twice a year, at the maximum. It is not a common event, nor it is realized from one day to the next. As soon as the community determines there are several deaths, they obtain the services of a *Nele* who, through his visions, interprets the reason for the deaths. The smoke of the pipe can last 1 or 2 days. Afterwards, the community must wait 4 to 8 days to know the results of the ritual.

Another ritual is the aromatic smoking of the homes with pepper, which does not require the convocation of the whole community nor the presence of the *Nele*. Besides these two rituals, there are other practices not related specifically to malaria, but which influence vector control in the communities. The ritual of the drinking of the *Chicha* is performed in various festivities within the Guna culture, whether a wedding, a cutting of a girl’s hair, or a puberty celebration (named *Surba Inna*, a ritual of female initiation which is practiced in Madungandi communities).

### Social connotations of malaria within Guna culture

**Fig. 4:**
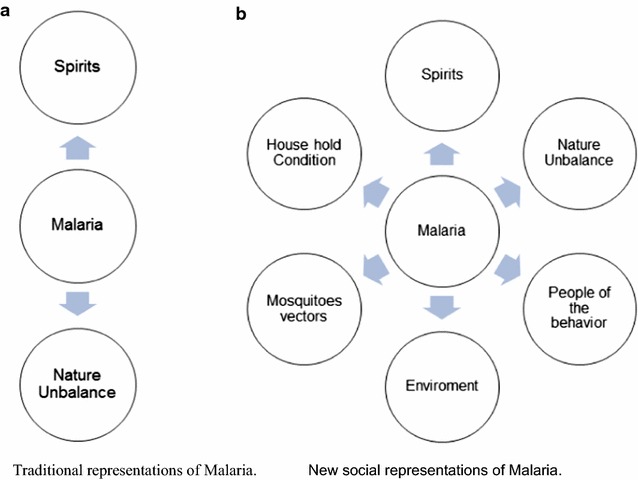
Reconfiguration of social representation of malaria in the Guna culture. **a** Representation of the constitution of traditional social representation of malaria by the Guna of Madungandi. **b** New social representation of malaria by the Guna of Madungandi

The Gunas consider malaria presence as an imbalance between nature and spirits. In this study, it was possible to develop a new social representation for malaria (Fig. 4). In approaching the interpretative field of the disease in Guna culture, relaying a representative frame, malaria as a disease is conceived as exogenous, with external variables and not controlled by humans; these variables are magic, mystic and spiritual. To manage this spiritual world, establish order and health within the population, the Guna need the assistance of the *Nele* and *Absogedis*, which are empowered to tie malignant spirits through their chants and rituals of smoking the pipe.

By being conceived as an external disease, malaria and the transmitting mosquito, as an element of nature; preventive actions are not taken within this population. It is perceived as an insuperable reality that mosquitoes have always existed and will always be present. The communities are not convinced that they are able to initiate changes to control this disease. The control is not within them but within the protective spirits and the traditional doctors. Death from malaria does not motivate to conduct change, given that death is conceived as an expected fact that must happen, and where humans have no control.

This new representation of malaria is part of the identity of an indigenous people and following their own ethnic development, they will identify the need to modify such belief. The health system can contribute with necessary information and work alongside the community so that the population might understand that despite the magic-religious component, this disease has a behavioural component with which they can keep control. The health system should utilize culturally adapted methodology to achieve empowerment of the population to prevent and control malaria.

### Strategies for an intercultural approach

As for strategies for an intercultural approach to malaria, the opinions of the women were diverse. Some manifested that nothing could be done, as was indicated by the following participant:

*“I believe nothing can be done because is useless. I have seen people who tried to talk but to no avail because our tradition is there. In so many years that I have seen and worked in medical tours, nothing can be done because the authorities close themselves up and say no.”* Focus group of women from Akua Yala.

To achieve an intercultural approach, it is important to have good coordination, not only with traditional doctors, but with traditional authorities as well.

*“Sometimes traditional medicine and modern collide with the date. That is why I tell you….I do not like this situation. It bothers me to be fighting with my companions due to this situation.” Sahila* of Icanti.

An intercultural approach will include a change of conduct which will incorporate environmental hygiene measures to promote the elimination of mosquito breeding places. When the women were considered for these prevention actions, they were asked what was needed for them to obtain a major involvement of the community to eliminate mosquito breeding places. They answered:

*“We need to have authority with a strong hand to demand that the community obey and clean their houses and its surroundings. There are authorities here, but they do not have this strength. They said it, but it has to come from within ourselves to want to clean. Women cooperate much more than men, and get organized better.”* Focus group of women from Akua Yala.

Several epidemiological, social, environmental, political and educational factors were identified in this study that might be related implicated transmission of malaria in the Guna population of Madungandi, Panama (Additional file 1). These factors should be considered by health authorities for an appropriate intercultural approach with the Guna population of Madungandi to achieve better results in malaria prevention and control.

## Discussion

In Panamá, malaria is the main parasitic disease transmitted by vectors, presenting a pattern of transmission focalized mainly in regions with indigenous populations; its incidence and morbidity causes a negative socio-economic impact. *P. vivax* generates more than 90% of the cases, with periodic outbursts of *Plasmodium falciparum*, mainly from cases imported from other countries [22]. In the indigenous regions, more than 85% of cases are diagnosed at the national level, representing 12% of the total population of the country [22]. Malaria is a challenge for public health professionals due to high level of mortality and morbidity in this area of the Americas [23].

In the Comarca Guna de Madungandi, malaria is transmitted in outbursts or peaks with variations associated with ecological and environmental conditions, and with climate and socio-economic activities. One of the most important factors is constant migration of native people and symptomatic and asymptomatic visitors from one site to another within the Comarca and from neighbouring Guna Yala Comarca, which determines the intensity and prevalence of malaria in this region of the country. Ongoing migration is an activity that is much associated with the Guna indigenous culture. The majority of locations are in proximity to sites that favour anopheline vectors, making them receptive and vulnerable to transmission of malaria.

The health system had faced varied and continuous difficulties in monitoring, prevention and control of malaria, especially within the Guna population and specifically in Comarca Guna de Madungandi. There is a great need to culturally adapt health guidance in the indigenous populations [24], which while adhering to international and national regulations needs to pay attention to holistic approaches, combining therapeutic resources of modern medicine and traditional indigenous medicine in the national health system [25, 26].

In the indigenous Guna population, health is understood to be the result of the integration of social-magic and biological factors [27]. The restoration of health in the Guna culture, as well as other indigenous cultures, is obtained through the use of traditional medicine and cultural practices. The concept of health-disease is attached to the culture of the Guna people, which means that the health process, disease and treatment, is conditioned to states beyond the body, and has to do with subjectivity and individual behaviour in the natural and social environment [28, 29]. This makes it difficult to arrive at an exhaustive definition of health from a transcultural perspective. However, it could be that the concept is wider than the absence of maladies and diseases within biological, psychological and social factors [30–32].

All the surveys about cultural aspects, both those related to malaria and to other diseases, present different findings, a fact that enhances the need to recognize the symbolic and conceptual factors that each society develops according to its experience, and which partly suggests the course that public health intervention should take [33].

Malaria is recognized by the Madungandi Gunas’ traditional medical system; its treatment is through ingestion and bathing with medicinal plants. In an interview about terms applied to malaria in Madungandi, it was called *boni cortiquit*, which means yellow fever, since they said that when you suffer from this disease the eyes turn yellowish. Other names given were *kui boni*, which means mosquito disease, *boni*, which means disease, and *wegueler*, which means fever [34]. However, malaria in the Guna population of Madungandi is not perceived as a problem, due to the co-existence that they historically have had with the disease. In their culture, the existence of other problems has priority, such as poverty, malnutrition, potable water, vomiting, diarrhea, and colds, among many other maladies.

Surveys performed indicate that communities do not consider the disease affects them because general socio-economic needs diminish health needs [35]. This demands reflection regarding the way programmes of public health have historically been administered in these communities. The presence of Guna woman in the home, a high percentage of whom suffer at least one episode of malaria, means they are more appreciative of health problems in the family. In the Ngäbe population, malaria is recognized within traditional medicine, acquires symbolic categories and has a place in the collective cultural construction of the disease [17].

Traditional medicine is used by a high percentage of the population of Madungandi. However, only 6% use it to treat malaria. Nevertheless, 78% perform traditional practices such as ‘Smoking of the Pipe’ and ‘Burning of the Cocoa and Pepper’ to prevent malaria [34]. Other indigenous groups of the country have specialists divided into several disciplines that are in charge of curing diseases through a direct relation with the spirits [22].

A specific case that reveals the existing issue between indigenous cultures and health systems was the case during an epidemic of *P.* falciparum malaria in 2004, where the rituals of protection against the disease performed by the *Neles* (the ceremony of the ‘Smoking of the Pipe’) were not understood and were interpreted by MOH as a rejection by the Guna of help in the emergency.

The health system’s attention to health services in indigenous people, aside from their main difficulties of geographic isolation, limited access to health services, poverty, and analphabetism, assumes the pertinence of the use of ethnographic and hermeneutic methods to produce and interpret the data [36]. The health services face difficulties related to the lack of an adequate approach by health personnel to cultural differences, language barriers, communication, lack of comprehension of the beliefs, customs, traditions, and values of the indigenous populations [26].

Previous surveys of the cultural domain surrounding malaria are an important element in the success of control of the disease in different parts of the world. For example, in Sucre State, Venezuela, this knowledge and the focus employed by sanitary personnel reduced malaria incidence [37]. In Burkina Faso, comprehension of social representations about malaria allowed the use of insecticidal bed nets to be implemented to control the disease in communities [38]. In Ivory Coast, communities do not accept the use of diagnostic procedures that involve taking of blood due to HIV; to know this led to a change of strategies for the community [39]. Knowledge of cultural aspects was important in these experiences, and the findings differ according to communities, which enhances the importance of studying them at local level.

In Panamá, the main barrier to prevention and control programmes of malaria, has historically been of cultural nature. A basic problem has been linguistic differences between indigenous and non-indigenous population groups [40]. Traditional medicine plays an important role: sometimes rituals are executed and coincide with the actions of monitoring and control by health personnel. There are potential indicators tied to cultural factors of the population, such as language, migratory patterns, practices and rituals. The lack of effective control by malaria programmes in regions with indigenous populations is mainly due to lack of comprehension and lack of indigenous cultural empowerment. Public health programmes are certainly overwhelmed due to structural problems of social inequity which give rise to an environment of unsatisfied needs, which irretrievably affect the process of health and disease [41]. Sanitary programmes are designed without the participation of involved communities, as well as a lack of knowledge about local beliefs regarding malaria and of socio-political dynamics, which means control measures are imposed out of context, thereby reducing coverage and impact of interventions [42]. The design of strategies for malaria control in the context of a programme with several interventions demands knowledge of the involved communities, not only from a classic epidemiological perspective, but also of the people’s collective knowledge about the disease and its treatment [9, 43].

## Conclusion

The findings of this survey about the social connotations of malaria can significantly contribute to prevention and control of malaria programmes through new variables not previously considered, to facilitate an intercultural approach to improve the perception of the Guna population. To be successful, policies and health programmes must be established according to the reality and cosmovision of the Guna indigenous culture about malaria. It is necessary to perform an intercultural approach which guaranties the sustainability in time of anti-malarial intervention measures. While this continues to be ignored, the indigenous populations will be at risk of transmission and prevalence of malaria in their region and the rest of the country.

## Additional file

**Additional file 1.** Epidemiological, socio-economical, environmental, behavioral and political factors associated with the transmission of the malaria in the Guna population of Madungandi, Panama.

## Abbreviations

NMCP: National Malaria Control Programme Malaria
MOH: Ministry of Health
masl: metres above sea level
GCMC: General Congress of the Madungandi Comarca
GOM: Guna Organization of Madungandi
