# Supplementary material for: Social representations of malaria in the Guna indigenous population of Comarca Guna de Madungandi, Panama
Source: Malar J. 2017 Jun 15;16:256. doi: 10.1186/s12936-017-1899-4 (PMC5472999; doi:10.1186/s12936-017-1899-4)
Supplement: Supplementary file 1 — Additional file 1. Epidemiological, socio-economical, environmental, behavioral and political factors associated with the transmission of the malaria in the Guna population of Madungandi, Panama. [file 12936_2017_1899_MOESM1_ESM.doc]

**Additional file 1. Epidemiological, socio-economical, environmental, behavioral and political factors associated with the transmission of the malaria in the Guna population of Madungandi, Panama.**

| **Key Factors** | | **Evidences** | **Hypothesis** |
| --- | --- | --- | --- |
| **Epidemiological** | |  |  |
| **Epidemiological surveillance** | Limited spatial and temporal coverage in the active search of suspicious cases in the different sites of the Madungandi comarca. | Lack of personnel and budget. | Lack of an increase in budget and technical personnel culturally trained affects the efficacy of control measures against malaria. |
|  | Difficulty in following anti-malarial treatments and the registration of outbursts in vulnerable communities due to the cultural habit of migration. | Outbursts registers. | The cultural habit of migration of the indigenous gunas become a risk factor for the malaria distribution. |
|  | Lack of involvement of the traditional doctors in the monitoring and treatment of malaria. | There is not a program to involve the traditional doctors in the prevention, monitoring and control of malaria. | The involvement of the traditional doctors could improve significantly the prevention, monitoring and control of malaria. |
| **Entomological**  **surveillance** | The strategy of integral management of vectors is not applied. | A program for the integral management of vectors is non-existent. | The integral management of vectors can establish a strategy of malaria anti vector control much more effective. |
|  | Lack of coverage and reticence from the population to the application of the residual spraying in the houses. | Statistical registers. | The reticence to the in-houses spraying contributes to the increase of malaria transmission by the vectors mosquitoes. |
|  | Environmental interventions with the community involvement to eliminate the breeding places are not carried out. | There is not a program of anti- vector fight with community participation. | The community participation is fundamental in the application and maintenance of strategies for the anti- vector fight. |

| **Socioeconomic and Cultural** | | | |
| --- | --- | --- | --- |
| **Access to health facilities** | Limitation in the time for the appropriate diagnosis and treatment, and the active and passive search of suspicious cases due to geographical coverage. | Registration of time of diagnosis and treatment of malaria cases. | Diagnosis and timely treatment Is fundamental for malaria control. |
|  | Majority of the population does not attend the health facilities due to the distance and lack of money for their transportation. | Small attendance to the health facilities of the indigenous guna population. | The attendance to the health facilities by the population is important for the prevention, monitoring and control of malaria. |
| **Housing conditions** | Gunas houses due to their type and their construction materials favor the entrance and exit of the vectors mosquitoes. | Type of housing within the guna culture. | The type of guna housing is a risk factor for the transmission of malaria. |
|  | Houses built near to anopheline vectors mosquitoes breeding sites. | Socio-environmental register. | The houses near to vectors mosquitoes breeding sites are more vulnerable to the malaria transmission. |
|  | Limited distribution of mosquito nets and reluctance to the application of the in-houses spraying with residual action insecticides. | Register of the spraying coverage and lack of distribution of mosquito nets. | The lack of sanitary education about the use of mosquito nets and in-houses spraying bears on malaria control. |
| **Transmission Risk (susceptible contact/vector)** | The indigenous communities are generally situated very close to rivers or lakes where the anopheline vectors breeding places are found. | Cultural habit of the indigenous population. | If a program to improve the socio-environmental conditions and the elimination of breeding places is implemented, the transmission of malaria is reduced. |
|  | The constant migration limits the monitoring, prevention and control of the disease. | Statistical registries. | Coordination between the traditional indigenous authorities and the health ones is important for the malaria control. |
|  | Changes in soil use and new humane settlements generate ecological and socio- environmental changes which favor the proliferation of anopheline breeding sites and the risk of transmission. | Surveys of breeding sites during the outbursts. | The bad use of the soil could increment the number of breeding sites and the malaria transmission. |

| **Environmental and Behavioral** | | | |
| --- | --- | --- | --- |
| **Environmental**  **Conditions** | The gunas communities are situated in places that present ecological and socio- environmental conditions favorable for the presence of anopheline breeding sites. | Technical reports. | The location of the communities next to mosquitoes breeding sites increase the risk of malaria transmission. |
| **Change of use of the land** | The deforestation increment for agricultural activities, new human settlements, indigenous cultural practices, favor the risk of transmission and establishment of malaria. | Technical reports. | The inappropriate use of the land favors the increment of mosquitoes breeding sites. |
| **Knowledges** | A survey among the guna indigenous population of Madungandi, showed a moderate knowledge about the malaria (causes, symptoms, treatment and prevention). | Scientific article. | The sanitary education culturally adapted can obtain a better prevention, monitoring and control of the malaria. |
| **Appraisal of the prevention and control of the malaria** | Lack of a change of attitude and practices about the prevention and control of malaria. Limited use of mosquito nets, reluctance to the spraying of houses with insecticides and abandonment of the antimalarial treatment. | Lack of sanitary education programs in a permanent fashion. | The sanitary education culturally adapted can bring about a change of attitude among the indigenous population about the malaria. |
| **Social Participation** | Little social participation of the population in the measures of prevention and control of the malaria at the individual level and organized community groups. | Lack of programs for community participation. | The community participation can improve and enhance the efficiency and maintenance of the anti-malarial strategies. |

| **Education** |  |  |  |
| --- | --- | --- | --- |
| **Education and Awareness Raising** | Insufficient or null culturally-adapted sanitary education activity about monitoring, prevention and malaria control. | Lack of sanitary education programs. | The sanitary education can improve and enhance the efficiency and maintenance of the anti-malarial strategies. |
| **Cultural Practices** | The lack of communication and joint coordination between the indigenous traditional authorities and the ones from the health ministry about the use of cultural practices like “Pipe Smoking”, “Burn of Cocoa”, traditional medicine and migration, limit the monitoring, diagnosis, treatment and follow-up of the malaria cases. | There is not an on-going coordination and communication between the indigenous traditional authorities and the ones from the health ministry. | The coordination between the traditional indigenous authorities and the ones from the health ministry is important for malaria control. |
| **Volunteer collaborators** | Lack of a program to involve volunteer partners in the indigenous communities. | There is not such a program of volunteer partners. | The volunteer collaborators are necessary for the monitoring, prevention and control of malaria. |

| **Policies and Programs of Indigenous Health** | | | |
| --- | --- | --- | --- |
| **Regulations and Programs** | Absence of a program of intercultural sanitary education for the health workers.  Absence of programs and intercultural approach health plans in the indigenous populations. | Weakening of the indigenous sanitary program. | The improvement of the “Dirección de Asuntos Sanitarios Indigenas”- (Department for the Indigenous Sanitary Issues)-will help to improve the health conditions in the indigenous populations. |
|  | Little coordination between the actions of the Dirección de Asuntos Sanitarios Indigenas with another health programs. |  |  |
| **Financial and Economic Resources** | It is necessary to improve the intervention measures by the malaria program at the technical-operative level, logistic, structural and financially in order to maintain and adequate answer and culturally adapted to the actual conditions of transmission, which produces a weakening of the operative actions, without the quality, coverage, intensity and needed frequencies. | Lack of financial and economic resources. | The budget increase to the sanitary programs will help to reduce the impact of malaria in the indigenous populations. |
| **Investigation and Intercultural Approach** | It is necessary to perform surveys about the guna indigenous culture in order to obtain an attitude change that will allow the communities to know the origin of the health problem and be able to implement measures for its solution. | Only a few surveys about the malaria within the indigenous cultures in Panamá. | The technical and scientific surveys through an anthropological and sociological approach will help to improve the policies, programs, and health strategies in the indigenous populations. |
|  | Difficulty in following-up the anti-malarial treatments and register of outbursts in vulnerable communities due to the lack of understanding of the cultural practices. |  |  |
|  | The majority of the guna populations use the traditional medicine; due to their importance within the culture, Neles, Innatuledi, Absogedis, must be involved in the prevention, monitoring, and malaria control. |  |  |
